# Early career demanding psychosocial work environment and severe back pain and neck/shoulder pain in experienced nurses: A cohort study

**DOI:** 10.1177/14034948231151992

**Published:** 2023-02-22

**Authors:** Tobias Sundberg, Eva Skillgate, Petter Gustavsson, Ann Rudman

**Affiliations:** 1 Musculoskeletal and Sports Injury Epidemiology Center, Department of Health Promotion Science, Sophiahemmet University, Sweden; 2 Institute of Environmental Medicine, Karolinska Institutet, Sweden; 3 Department of Clinical Neuroscience, Division of Psychology, Karolinska Institutet, Sweden; 4 School of Health and Welfare, Department of Caring Sciences, Dalarna University, Sweden

**Keywords:** Work environment, nursing, back pain, neck pain

## Abstract

**Aims:**

Back pain and neck/shoulder pain are common among nurses. The aim of this study was to investigate the association between nurses’ exposure to a demanding psychosocial work environment during the first three years after graduation and the occurrence of severe back pain and neck/shoulder pain in the longer term, 11–15 years later.

**Methods:**

The Longitudinal Analysis of Nursing Education (LANE) study (nursing graduates from 26 Swedish universities in the years 2002, 2004 and 2006) was used to create two risk cohorts of nurses not reporting severe back pain (*n*=1764) or neck/shoulder pain (*n*=1707). Nurses exposed to a demanding psychosocial work environment for one, two or three of the first three years in their career were compared to nurses not having a demanding psychosocial work environment for any of these three years regarding the incidence of severe back pain or neck/shoulder pain at follow-up, 11–15 years later. Relative risks (RR) with 95% confidence intervals (CI) were calculated using binomial regression.

**Results:**

The RR (95% CI) of having severe back pain for nurses who had a demanding psychosocial work environment for one of the three years was 1.36 (0.82–2.28) and 2.08 (1.21–3.57) for two of the three years and 2.82 (1.43–5.55) for all three years. Corresponding RRs (95% CIs) for severe neck/shoulder pain were 1.35 (0.87–2.10), 1.49 (0.88–2.51) and 1.41 (0.62–3.20), respectively.

**Conclusions:**

**Nurses who were repeatedly exposed to a demanding psychosocial work environment early in their career reported severe back pain to a higher extent in the longer term.**

## Introduction

Back pain, typically reported as non-specific pain in the lower part of the spine, is a very common and costly musculoskeletal disorder of public health concern globally [1], as is pain in the upper part of the spine in the neck/shoulder area [2]. Work-related musculoskeletal disorders are frequent in nursing, and nurses may be especially susceptible to musculoskeletal pain in the back, neck and shoulders [3–6].

Among several musculoskeletal disorders affecting nurses, back pain has been reported to have the highest cumulative incidence as well as the highest prevalence of persistent or recurrent pain, which may influence nurses’ abilities to perform functional task and their work abilities [7]. A previous investigation, from the Longitudinal Analysis of Nursing Education (LANE) study [8] which monitored nursing students from their last year of studies to one and two years after their graduation, reported that this target group had persisting high prevalence of back pain (about 40%) and neck/shoulder pain (about 50%) [9]. Notably, the LANE study has a unique representation of nursing students by including participants from any of the 26 Swedish universities providing training for nurses at the time of the study [8].

Systematic research targeting nursing personnel has reported associations between high psychosocial demands/low job control and prevalent and incident back pain, prevalent shoulder pain as well as pain at other anatomical sites [10]. Further, low social support has been associated with incident back pain, and effort/reward imbalance has been associated with prevalent musculoskeletal disorders in any body region [10]. Similarly, systematic review findings have shown that exposure to certain occupational psychosocial factors among nursing staff, such as high demands, may be significantly associated with pain or discomfort in the neck, and that work-related imbalance between effort and reward can be associated with pain or discomfort at any anatomical site [11]. Taken together, these previous findings suggest that work-related psychosocial factors are associated with musculoskeletal disorders, including back and neck pain, among nursing staff. Identifying risk factors that are associated with the occurrence of back and neck pain may constitute the base for conducting relevant studies investigating preventive strategies in this domain, and to optimise demanding working environment for nurses, which should be encouraged. To our knowledge there have been no previous studies investigating longer-term exposures and associations between a psychosocial demanding work environment and the occurrence of musculoskeletal pain in nurses. The aim of this study was therefore to further the understanding of the association between a demanding psychosocial work environment during the first three years after graduation and the occurrence of severe back pain and neck/shoulder pain in nurses in the longer term, 11–15 years later.

## Methods

### Ethics

All participants provided informed consent after having received oral and written study information, which included details about confidentiality measures and informing them that they could quit their participation at any time without any consequences. The study was approved by the Regional Research Ethics Committee at Karolinska Institutet, Stockholm, Sweden (Dnr 01-045) and the Regional Ethics Review Board in Stockholm, Sweden (Dnr 04-587, Dnr 2016/793-32).

### Design and setting

This was a population-based cohort study of nurses graduating and working in Sweden.

### Study population

**Figure 1.**
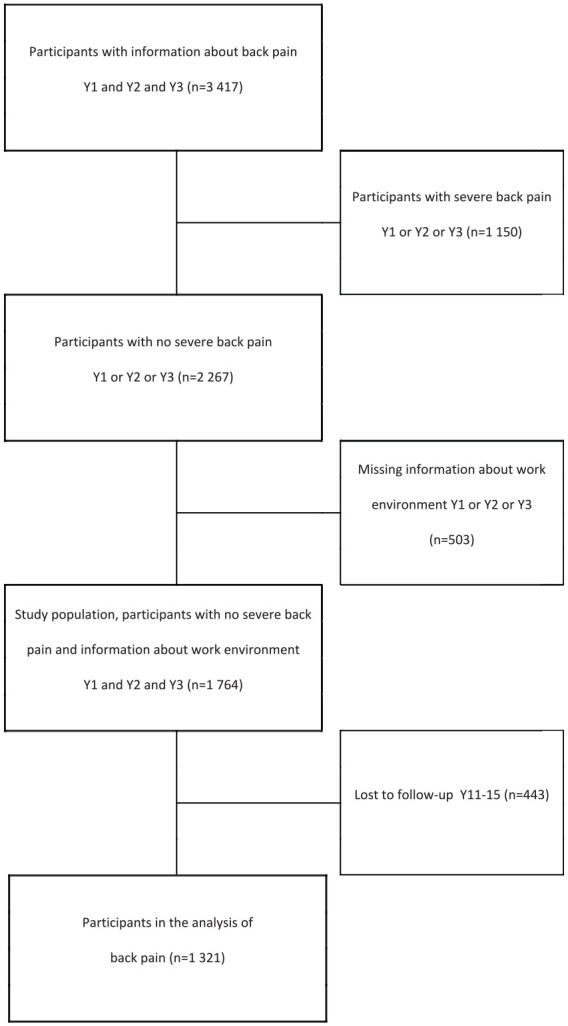
Flow chart of the process defining the study population in which back pain was studied. Y1: one year after graduation; Y2: two years after graduation; Y3: three years after graduation; Y11–15: follow-up 11–15 years after graduation.

**Figure 2.**
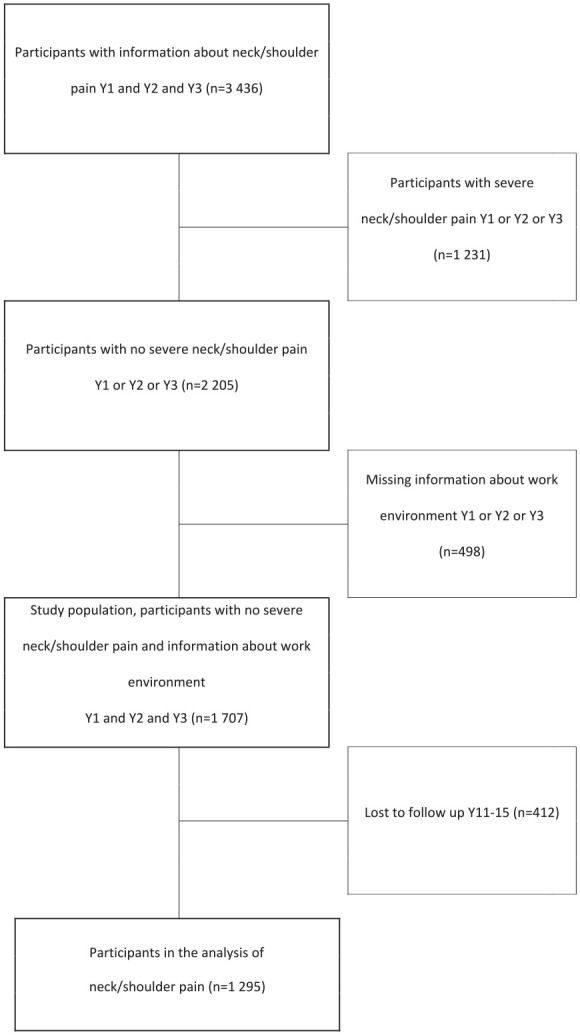
Flow chart of the process defining the study population in which neck/shoulder pain was studied. Y1: one year after graduation; Y2: two years after graduation; Y3: three years after graduation; Y11–15: follow-up 11–15 years after graduation.

The LANE study cohorts (nursing graduates from 26 universities in Sweden in the years 2002, 2004 and 2006) [8] were used to create two risk cohorts: nurses *not* reporting severe pain in the back (*n*=1764; Figure 1) and nurses *not* reporting severe pain in the neck/shoulder (*n*=1707; Figure 2) during any of their first three years of working life. By creating these ‘healthy’ risk cohorts of nurses, we were able to study the effect of repeated exposure to a demanding work environment regarding the incidence of severe pain in the back and neck/shoulder in the long term (11–15 years later). The flows of participants in the creation of the cohorts are presented in Figures 1 and 2, which also show that the proportions of participants who could be followed for the long-term follow-up after more than a decade were 75% (1321/1764) for severe back pain and 76% (1295/1707) for severe neck/shoulder pain.

### Data collection and measurements

Data for this study were collected using questionnaires among the nurses once per year during the first three years after graduation (considered as the baseline in this study) and once 11–15 years after graduation (the follow-up). All data were based on self-reported items, including the prevalence of pain in different body areas, work-related factors and background variables of demographic characteristics. Statistics Sweden provided technical, language and data-collection support. Back pain and neck/shoulder pain were assessed using two single items from a physical symptom checklist. The physical symptoms checklist was designed for the LANE study to assess somatic illness, and the question was phrased ‘During the last four weeks, have you experienced the following health problem?’ Responses were given on a four-point scale, with the options 0=‘none’, 1=‘mild’, 2=‘moderate’ and 3=‘severe’, measured during the last four weeks. Prevalence at baseline (to define the risk cohorts) and the occurrence 11–15 years after graduation of severe back pain and/or neck/shoulder pain (the outcomes in the explanatory analyses) was defined as a positive response to the response category ‘severe’.

The operationalisation of the ‘demanding psychosocial work environment’ was based on the participants’ answers to questions about work context and situation and occupational-related psychosocial factors. Specifically, the definition of the demanding work environment profile was characterised by a high degree of quick decisions, complex decisions, fast work pace and many emotional decisions, as well as a low degree of influence, appreciation and participation in their work. The occupational psychosocial working conditions utilised as measures of exposure were assessed with eight items: seven items from the General Nordic Questionnaire for Psychological and Social Factors at Work [12] and one item from the Copenhagen Psychosocial Questionnaire [13]. Responses were given on a five-point scale, with the options 1=‘very often or always’, 2=‘often’, 3=‘sometimes’, 4=‘rarely’ and 5=‘very rarely or never’. In order to capture stable characteristics of the work environment early in the career, the scales were converted into categorical indicators, where categories ‘very often or always’ and ‘often’ were coded as 1, and categories ‘sometimes’, ‘rarely’ and ‘very rarely or never’ were coded as 0. Exposure to a demanding psychosocial work environment was empirically defined based on results from a latent class analysis calculating models separately estimated for each time point [14].

### Data analysis

**Figure 3.**
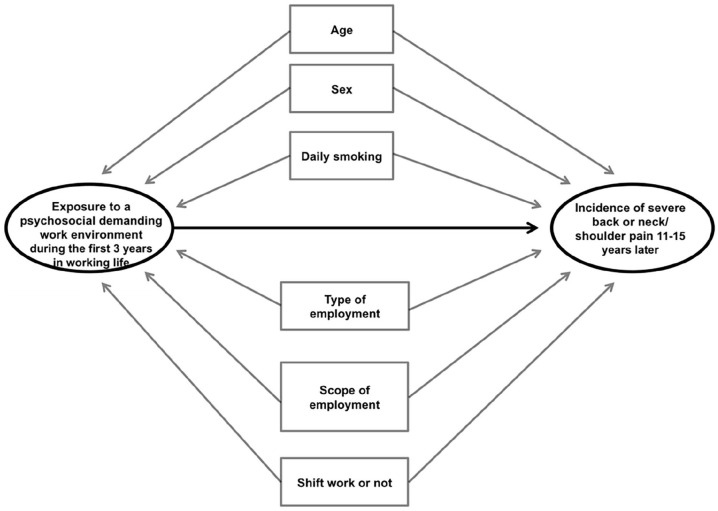
Directed acyclic graph diagram outlining the potential confounding factors taken into account in the analyses: age, sex, daily smoking, type of employment (permanent employment or other type of employment), scope of employment (full-time/part-time) and shift work or not.

The statistical analysis plan was approved by the authors before analyses began. Nurses categorised as having a demanding psychosocial work environment at baseline one, two or three of the first three years in their working life (exposed) were compared to nurses not having had a demanding psychosocial work environment any of the three years (reference group) regarding the occurrence of severe back pain or neck/shoulder pain at follow-up 11–15 years later with binomial regressions to calculate the relative risk (RR) of the occurrence of pain with a 95% confidence interval (95% CI). The following potential confounding factors, derived by the participants’ answers to the corresponding survey questions, were considered in the explanatory analyses based on a careful consideration of confounders, mediators and colliders by drawing a directed acyclic graph (Figure 3): age, sex, daily smoking, type of employment (permanent employment or other type of employment), scope of employment (full-time/part-time) and shift work or not. To consider further the confounding effect of the identified factors on the associations in the explanatory analyses, the factors were included, one at a time, into a crude model. If a factor changed the estimated risk ratio by ⩾10%, it was included in the final model. All *p*-values were two sided, and analyses were computed with STATA v15 (StataCorp, College Station, TX).

## Results

The majority (88%) of the nurses in the two risk cohorts were women, and the nurses’ mean age was 33 years at one year past graduation in both cohorts. The follow-up rate of the nurses was 75% (1321/1764) in the back pain cohort (Figure 1) and 76% (1295/1707) in the neck/shoulder pain cohort (Figure 2).

### Work environment and the association with the occurrence of severe pain

The results show that repeated exposure to a demanding psychosocial work environment, that is, to have such a work environment for two or three years, was associated with the occurrence of severe pain 11–15 years after graduation, especially in the back but also in the neck/shoulder in the long term, although the latter were not statistically significant (Table I). None of the potential confounders confounded the associations and were accordingly not adjusted for in the analyses. The RRs and 95% CIs of having severe back pain were 1.36 (0.82–2.28) for nurses who had a demanding work environment for one of the three years, 2.08 (1.21–3.57) for nurses who had such a work environment for two of the three years and 2.82 (1.43–5.55) for nurses who had such a work environment for all three years. Corresponding results for severe neck/shoulder pain were 1.35 (0.87–2.10) for one of the years, 1.49 (0.88–2.51) for two of the three years and 1.41 (0.62–3.20) for all three years.

**Table I.** Relationship between repeated exposure to a demanding psychosocial work environment during the first three years of working life after graduation and the occurrence of severe back pain and neck/shoulder pain, respectively, after 11–15 years.

None of the years (reference no demanding)		One of the years		Two of the years		Three of the years	
Exposed cases/all	Reference RR	Exposed cases/all	RR (95% CI)	Exposed cases/all	RR (95% CI)	Exposed cases/all	RR (95% CI)
*Cohort for analysis of back pain (*n*=1321)*							
29/621	1	26/408	1.36 (0.82–2.28)	21/216	2.08 (1.21–3.57)	10/76	2.82 (1.43–5.55)
*Cohort for analysis of neck/shoulder pain (*n*=1295)*							
39/622	1	34/401	1.35 (0.87–2.10)	19/204	1.49 (0.88–2.51)	6/68	1.41 (0.62–3.20)

RR: relative risk; CI: confidence interval.

## Discussion

This longitudinal study investigated the association between the exposure of nurses in Sweden to a demanding psychosocial work environment early in their careers, the first three years after graduation and the occurrence of severe back pain or severe neck/shoulder pain in the longer term, 11–15 years later. The main finding was that nurses who were repeatedly exposed to a psychosocially demanding work environment during the first three years in their working life had a significantly higher risk of having severe back pain 11–15 years later. The results also indicate a higher although not statistically significant risk of severe neck/shoulder pain than nurses who were not exposed to such a work environment.

### Discussion of findings

Back pain has been reported as a main type of persistent or recurrent category of musculoskeletal pain in nurses, affecting their functional task and working abilities [7]. Nurses’ work environment, commonly involving job tasks that include managing physical loads, might pose an increased risk for nurses acquiring musculoskeletal disorders [15]. Additionally, various psychosocial risk factors, such as high psychosocial demands and low job control, may impose associations with musculoskeletal disorders, including back pain and neck/shoulder pain, in nursing staff [10,11]. Accordingly, the demanding work environment of our study could be characterised by major psychosocial constraining factors, including a high degree of quick decisions, complex decisions, fast work pace and many emotional demands, combined with a low degree of work influence, appreciation and participation. These psychosocial factors and the current study findings align with the results of previous studies investigating risk factors associated with musculoskeletal complaints, including back pain and neck/shoulder pain, among nurses [3,10,11,16,17].

The period of most risk for back pain among nurses is not clearly understood. Previous studies suggest that back pain and neck/shoulder pain is already highly prevalent among nursing students during their undergraduate studies and that back pain may increase with the rise in work exposure after graduation [9,18]. Adding to those research reports, the current study, which to our knowledge is the first longitudinal study of the association between long-lasting exposure to a psychosocially demanding work environment early in nurses’ careers and the occurrence of severe back and neck/shoulder pain more than a decade later, suggests that it was mostly back pain, rather than neck/shoulder pain, that was associated with such exposure in the longer term. This is in accordance with previous findings that persistent or recurrent back pain, among other musculoskeletal disorders in nurses, seems to have the highest cumulative incidence and prevalence affecting nurses’ work abilities and their ability to perform functional tasks [7]. Further, the combined mentally and physically demanding work environments that nurses may experience, especially in some specialities such as surgery, might additionally cause work/family conflicts affecting back and neck pain in nurses [19]. Hence, it may be important to address occupational as well as family or social factors when considering interventions to manage musculoskeletal complaints of back and neck pain among nurses. Of further importance is that our results indicate that nurses free from severe back and neck/shoulder pain during the first three years in their career develop such pain later.

Despite repeated studies reporting findings of associations between psychosocial as well as physical factors and musculoskeletal disorders of back pain and neck/pain among nurses, which strongly suggest that major preventive strategies should be encouraged in this target group of health providers, there are still significant knowledge gaps on how best to prevent these ailments from manifesting [20,21]. So, what strategies and interventions should be prioritised to address demanding work environments and potentially prevent or limit back pain or neck/shoulder pain among nurses? Arguably, a demanding work environment that is characterised by major psychosocial risk factors is largely a challenge at the organisational level. Hence, providing selected physical or mental health interventions that are directed towards the individual nurse or groups of nurses that work in physically and emotionally stressful work environments might not be the most effective strategy to manage painful musculoskeletal or stress-related disorders in the longer term or to prevent staff turnover, albeit such interventions might provide a valuable management option in some cases [22–26]. Rather, it may be more effective to address organisational structures and processes to achieve better health outcomes for nursing staff [27], with recent research suggesting that the implementation of such supportive strategies might help facilitate a reduction in staff turnover [28,29].

Considering the current findings and the literature showing that, so far, there is very limited and inconclusive evidence from systematic research about the effectiveness of various interventions in the prevention and treatment of musculoskeletal disorders, including back and neck pain, among nurses [20,21], it may be hypothesised that by limiting repeated exposure to a demanding work environment for nurses early in their career, future severe back pain might be prevented. Accordingly, future studies that investigate strategies to support the development of optimal work environments for nurses at the organisational level, rather than testing isolated therapies or treatments for pain, are warranted. Future research should also target detailed descriptions about the nature of the back pain and neck/shoulder pain that nurses are experiencing in the longer term, as such findings may be of importance to inform the understanding and potential relevance of different strategies in the management of back pain and neck/shoulder pain experienced by nurses.

### Methodological considerations

The response rate of the long-term follow-up in the LANE study was 62%, which can be considered relatively high [8], as were the 75% and 76% follow-up rates of the back pain and neck/shoulder pain risk cohorts, respectively, in the current study. This, together with the prospective study design, where the exposure status was established before the occurrence of the outcome, suggests that the risk for selection bias in this study may be considered as low. Further, participants were largely representative of the Swedish nurses graduating in 2002, 2004 and 2006, which in turn, accounting for the nationwide recruitment of nursing students from all over Sweden to this study, might thus be considered representative [8].

The biggest threat to the validity of the results of a cohort study like this is that the risks of identified associations are affected by disturbance factors that coexist with the exposure and also cause severe pain in the back or in the neck/shoulder. If such confounding is present, incorrect conclusions might be drawn about the importance of the working environment for the occurrence of severe back or neck/shoulder pain. Accordingly, we took into account a number of potential confounding factors in the analyses, but none of those proved to be a confounder. However, there is still a risk that unmeasured and residual confounding may have affected the results, such as physical activity for which data were limited. There is also a risk that the methods used to measure work environment and to measure back and neck/shoulder pain are not optimal. To dichotomise the exposures into having or not having a certain work environment may misclassify important patterns of stress so that, for instance, nurses with considerable but not severe work-related stress are classified as not having a psychosocial demanding work environment. Such non-differential misclassification tends to dilute any associations, that is, the relationships reported in this study would be underestimated. Finally, there is a risk that the prevalence of a demanding work environment will be overestimated or underestimated if the participants who have chosen to be part of the study’s long-term follow-up differ from those who have chosen not to be included. In summary, we believe that the associations reported in this study are likely valid but potentially underestimated, even though the number of exposed cases are few in some analyses.

## Conclusions

The results suggest that nurses who are repeatedly exposed to a demanding psychosocial work environment early in their career report severe back pain to a higher extent in the longer term than nurses who are not exposed to such work environment.
